# Supplementary figures and images for: Predictability and transferability of local biodiversity environment relationships
Source: PeerJ. 2022 Aug 23;10:e13872. doi: 10.7717/peerj.13872 (PMC9415358; doi:10.7717/peerj.13872)

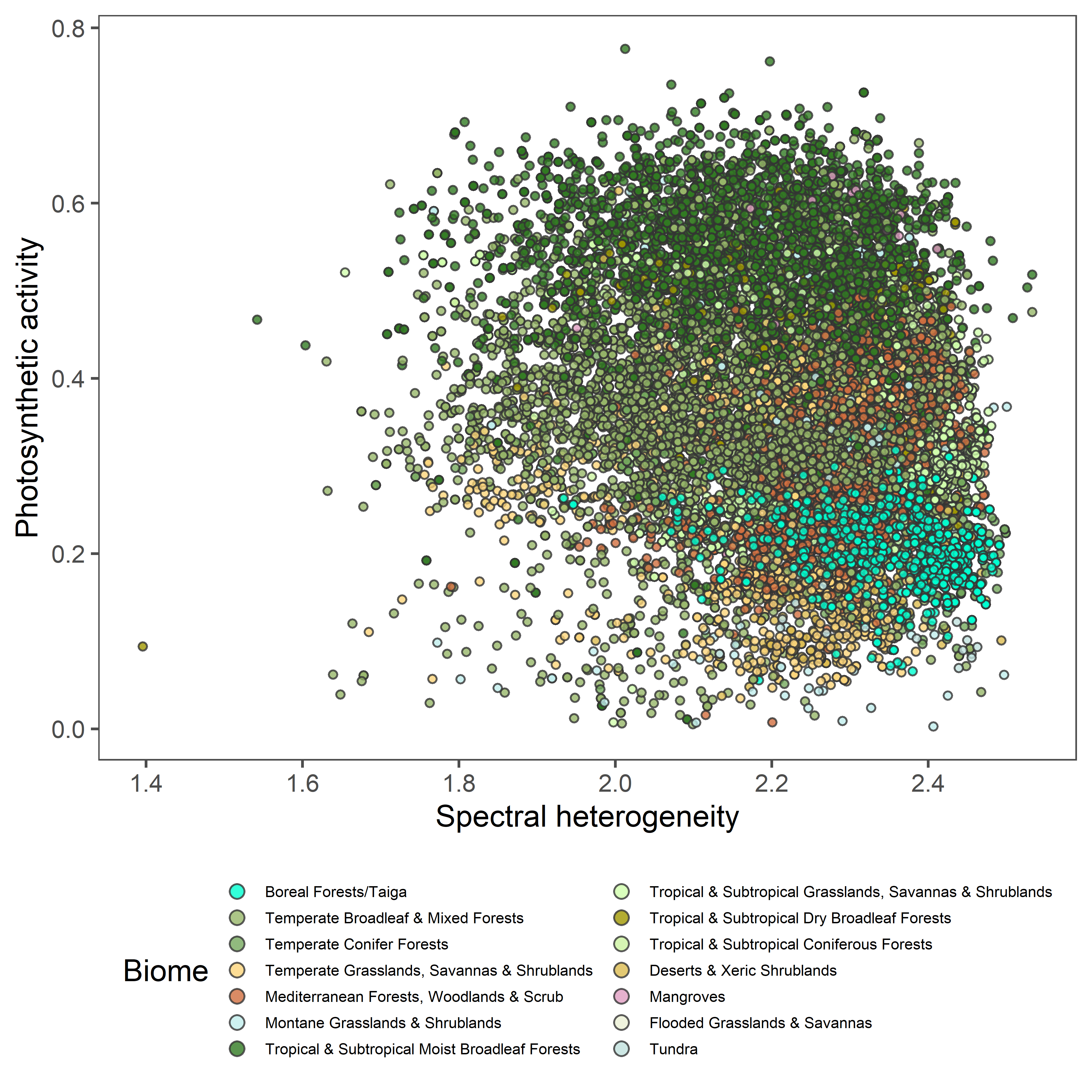

Supplement: Supplemental Information 1 — Sites coloured by Biome according to Dinerstein et al. (2017). [file peerj-10-13872-s001.png]

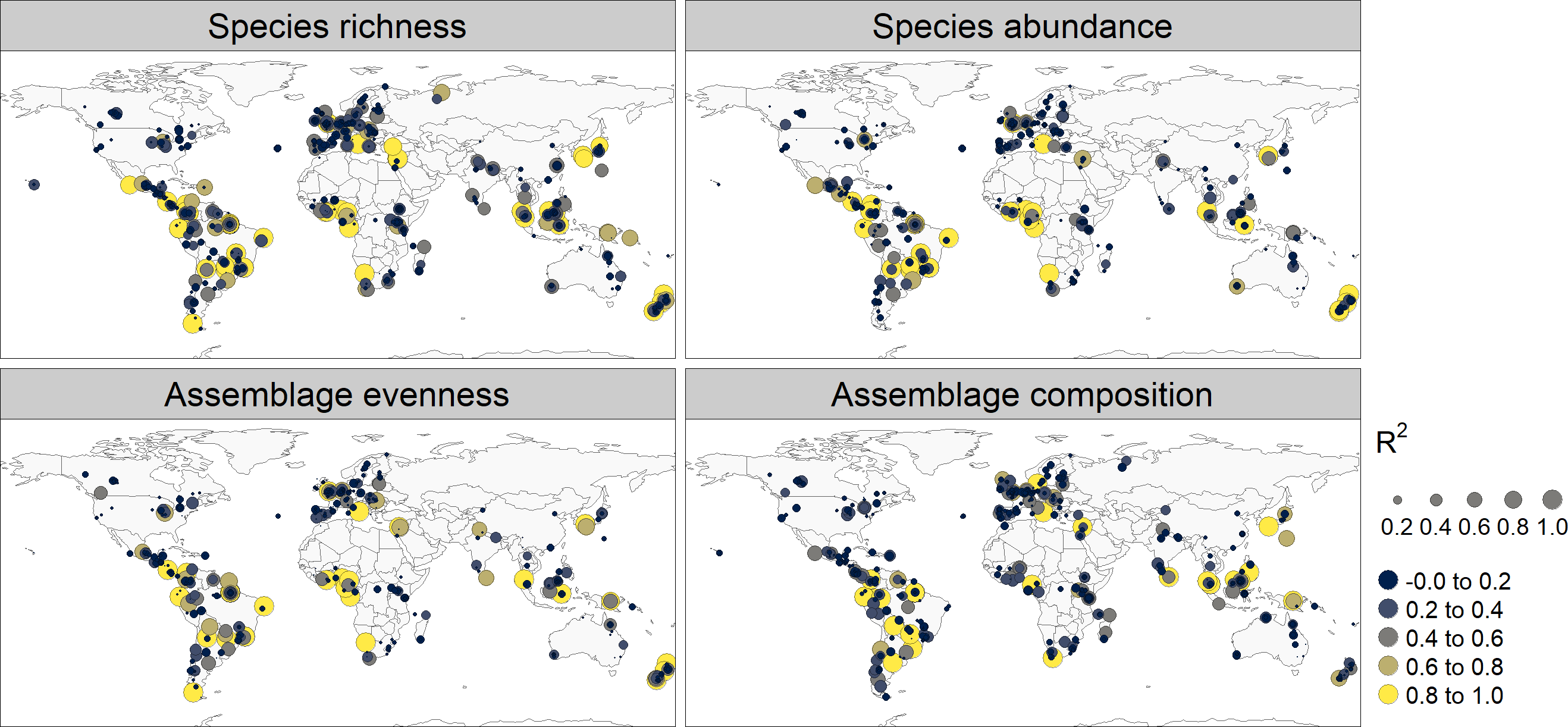

Supplement: Supplemental Information 2 — Each dot represents the centre coordinates of a study with size and colour indicating the explained variance. [file peerj-10-13872-s002.png]

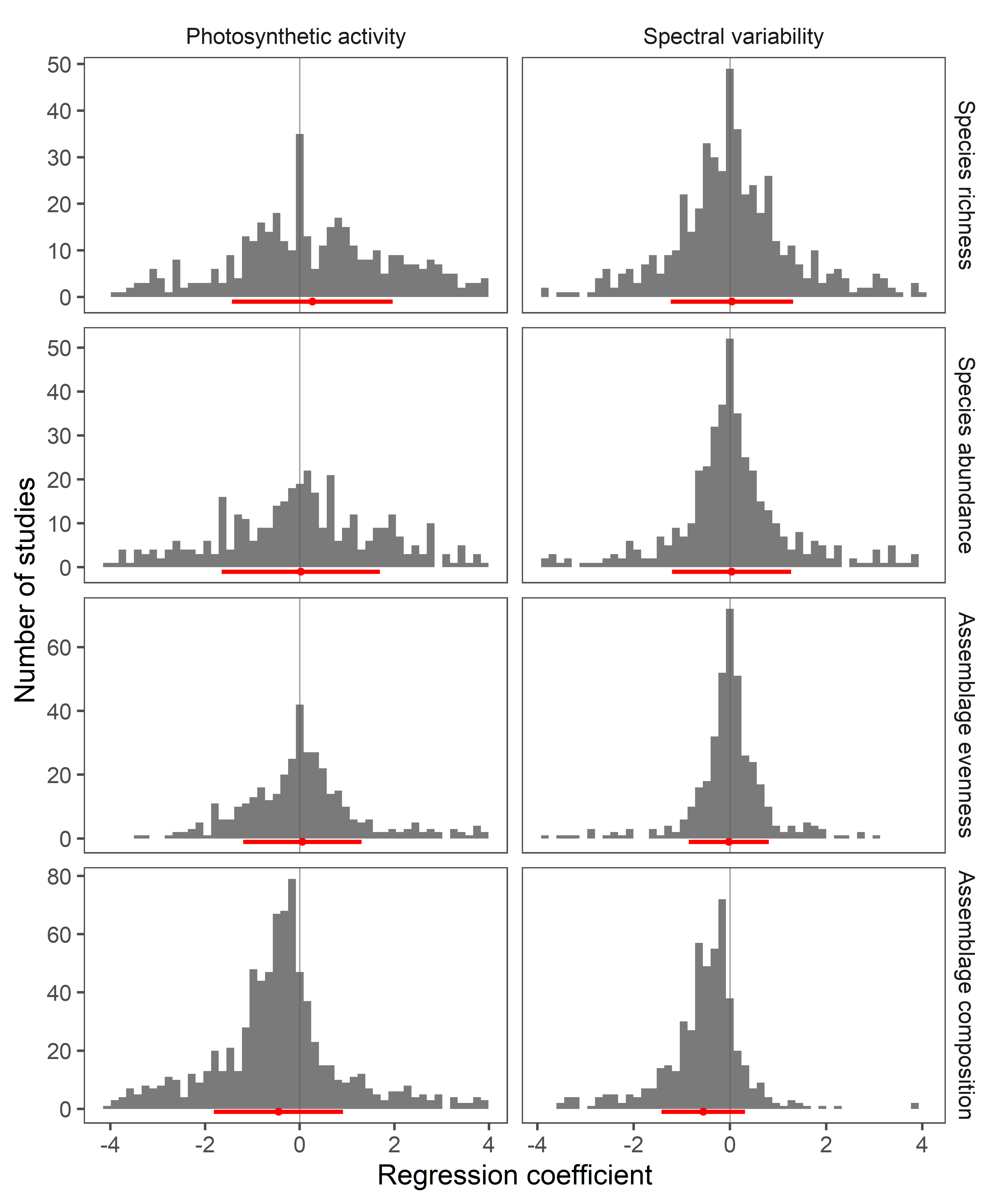

Supplement: Supplemental Information 3 — Error bars show the mean and 1 standard deviation of the regression coefficients. [file peerj-10-13872-s003.png]

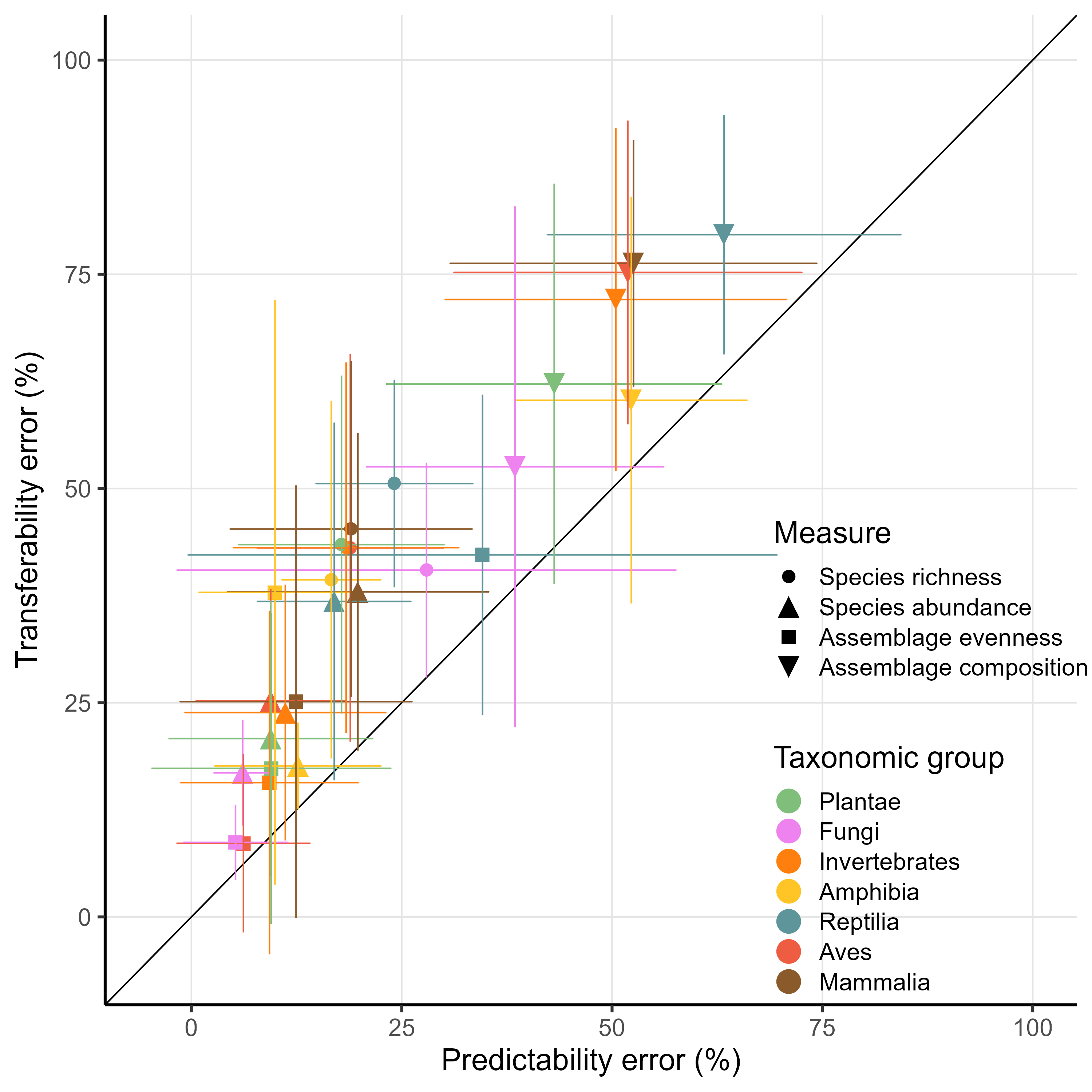

Supplement: Supplemental Information 4 — Colours and shapes as in Figure 4. [file peerj-10-13872-s004.png]

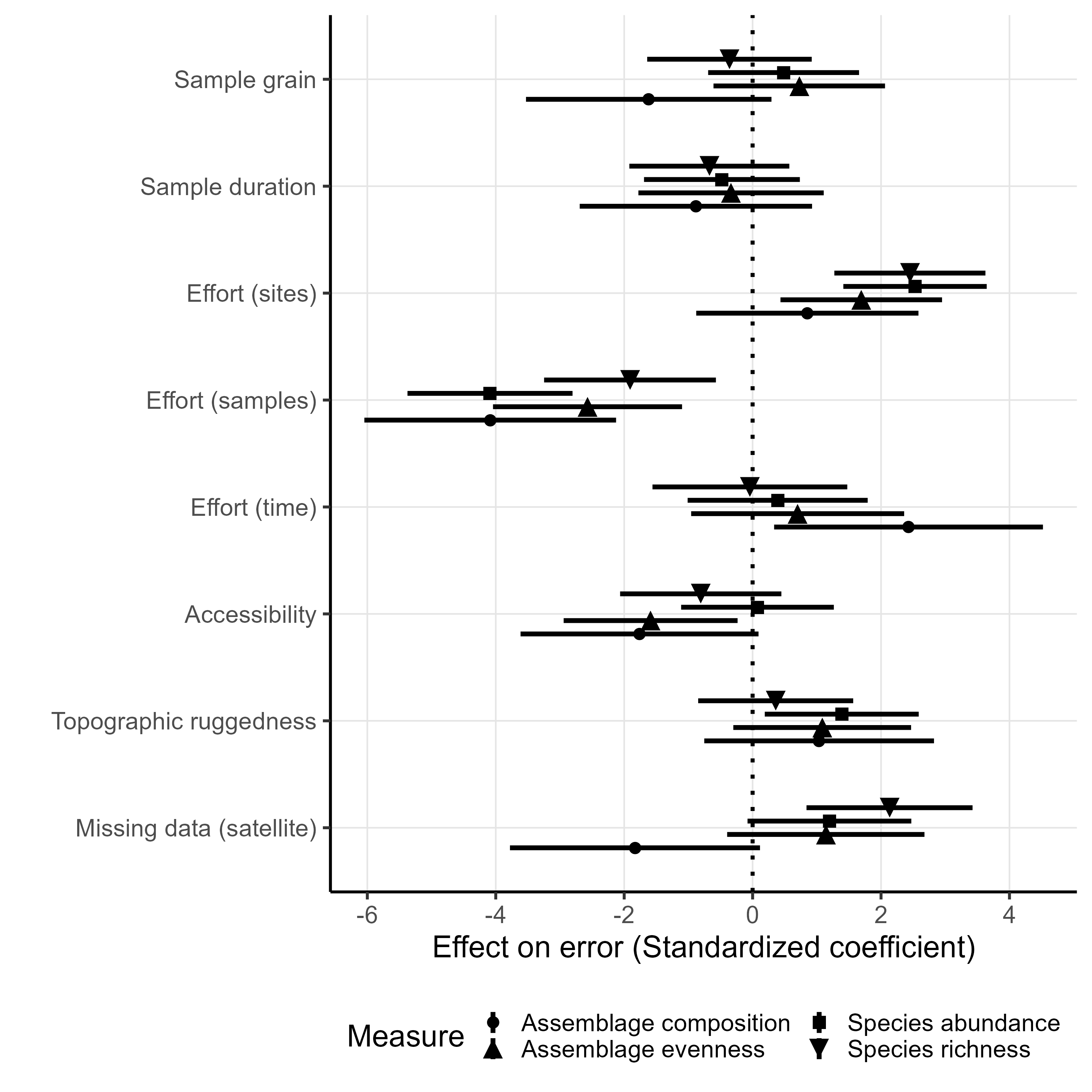

Supplement: Supplemental Information 5 [file peerj-10-13872-s005.png]

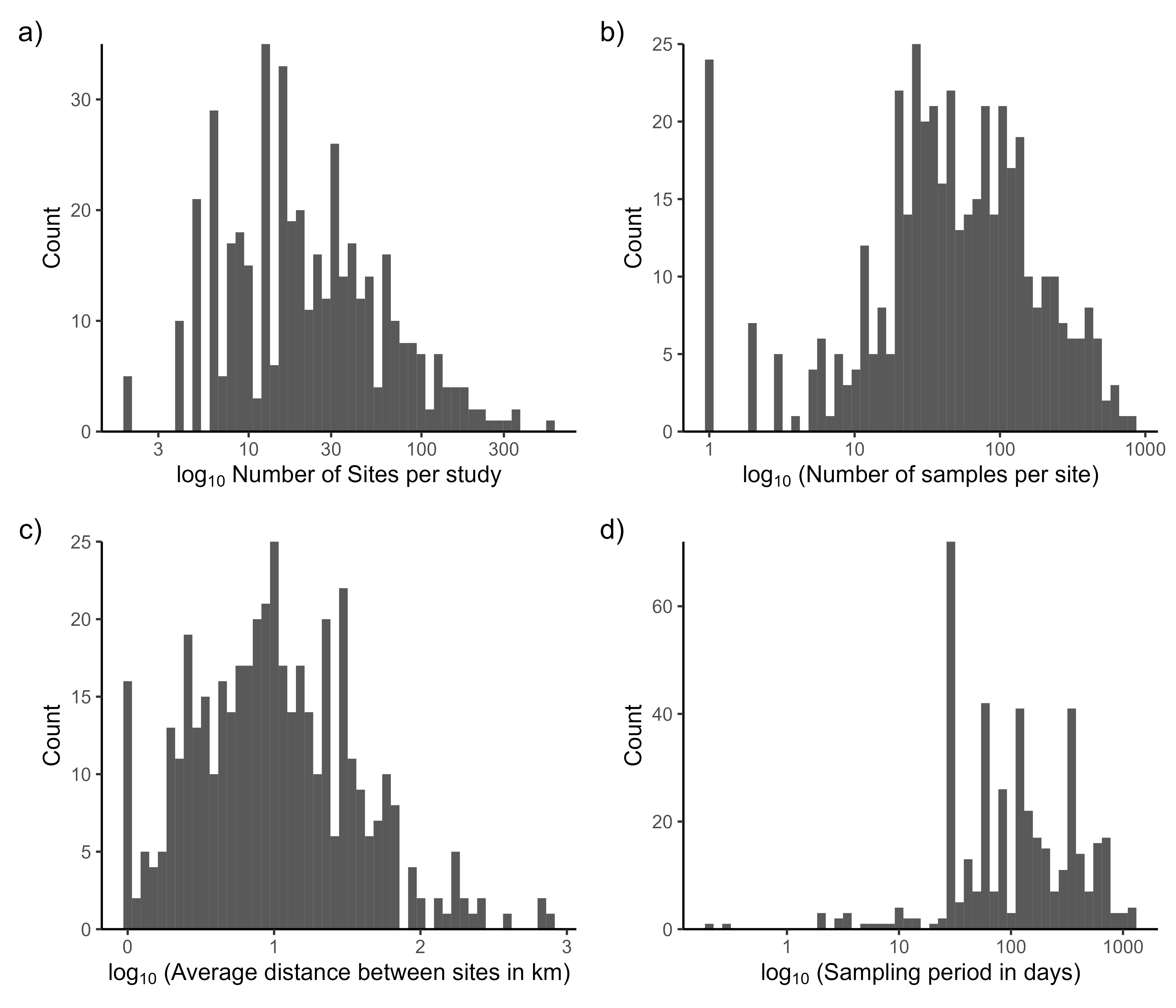

Supplement: Supplemental Information 6 — Shows the (a) log-transformed number of sites per single study, (b) log-transformed number of samples per site in a study, (c) log-transformed average distance between sites within a study and (d) log-transformed sampling period (start to end) in days. [file peerj-10-13872-s006.png]
